# Use of gadolinium-based contrast agents in head and neck cancer diagnosis, staging, and monitoring: current applications and future perspectives

**DOI:** 10.1007/s00330-025-12165-0

**Published:** 2025-12-13

**Authors:** Marco Parillo, Federica Vaccarino, Andrea Falzone, Elena Salvador, Fabio M. Doniselli, Carlo C. Quattrocchi, Àlex Rovira

**Affiliations:** 1 University of Verona, Verona, Italy; 2 Radiology, Multizonal Unit of Rovereto and Arco, APSS Provincia Autonoma Di Trento, Trento, Italy; 3 Department of Radiology, Hospital Universitario 12 de Octubre, Madrid, Spain; 4 Neuroradiology Department, Fondazione IRCCS Istituto Neurologico Carlo Besta, Milan, Italy; 5 Centre for Medical Sciences-CISMed, University of Trento, Trento, Italy; 6 Section of Neuroradiology, Department of Radiology, Hospital Universitari Vall d’Hebron, Autonomous University of Barcelona, Barcelona, Spain

**Keywords:** Gadolinium, Contrast media, Magnetic resonance imaging, Head and neck neoplasms, Practice guideline

## Abstract

**Abstract:**

Gadolinium-based contrast agents (GBCAs) have been fundamental to head and neck cancer (HNC) imaging, enabling effective detection, characterization, treatment response assessment, and disease progression monitoring of lesions. Additionally, perfusion-weighted imaging (PWI) utilizing dynamic contrast enhancement (DCE) has been evaluated for its ability to provide insights into microvascular parameters concerning blood flow within tumor tissue. Nevertheless, increasing worries regarding gadolinium accumulation within the central nervous system and its effects on the environment have led to a reconsideration of its application. This narrative review explores the current role of GBCAs in HNC imaging, the primary sequences used after GBCA administration, their interpretation, and potential alternative imaging approaches. Currently, GBCA administration is a cornerstone of multiparametric MRI for the diagnosis, staging, and monitoring of HNCs, commonly involving a 3D T1-weighted sequence with fat saturation during the equilibrium phase. While PWI shows potential for clinical application in HNCs, its broader clinical adoption requires further standardization. Notably, DCE can visually aid in detecting subtle tumors, and its application in the differential diagnosis of solid parotid lesions is yielding promising results. Arterial spin labeling is emerging as a compelling alternative for PWI, eliminating the need for GBCA administration. Other promising strategies for reducing or even avoiding GBCA use include hybrid PET/MRI examinations, the development of novel contrast agents (including high-relaxivity GBCAs and gadolinium-free contrast agents), and the implementation of artificial intelligence tools.

**Key Points:**

***Question*** *When should GBCAs be administered to patients undergoing MRI for HNCs?*

***Findings*** *GBCA injection is a cornerstone of multiparametric MRI for the diagnosis, staging, and monitoring of HNCs.*

***Clinical relevance**** GBCAs are recommended for HNC MRIs, with a possible exception for patients with no clinical or radiological evidence of recurrence after 27 months of follow-up. DCE is useful for identifying small carcinomas and characterizing parotid lesions*.

**Graphical Abstract:**

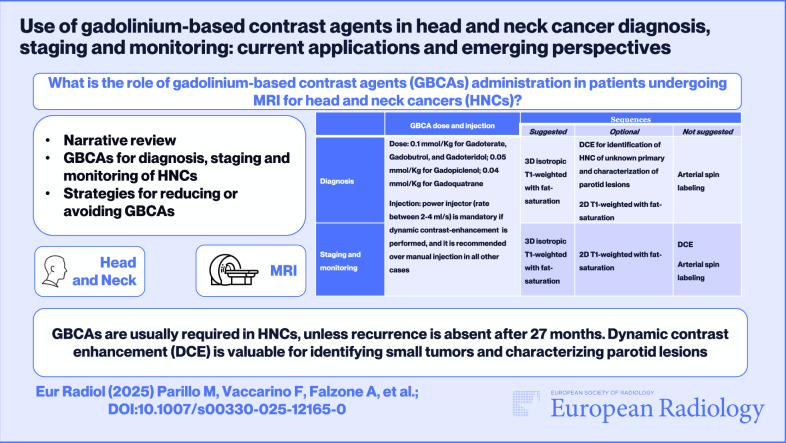

## Introduction

Head and neck cancers (HNCs) comprise a heterogeneous group of malignant diseases that can arise in different locations, such as the oral cavity, pharynx, larynx, paranasal sinuses, and salivary glands [1–4]. The estimated percentages of new cancer cases in the United States in 2025 for the main neck sites relative to all other cancers are: 1.86% for the oral cavity, 1.06% for the pharynx, and 0.64% for the larynx [5]. Over 90% of HNCs originate from epithelial cells, with squamous cell carcinoma (SCC) being the most prevalent type within this group [6].

MRI has become a crucial imaging technique for staging and monitoring HNCs, offering superior soft tissue differentiation compared to computed tomography (CT) [7]. Standard MRI protocols for HNCs include the intravenous injection of gadolinium-based contrast agents (GBCAs), which provide essential information on tumor vascularity and the degree of tumor spread that could not be apparent on unenhanced sequences (e.g., perineural or dural invasion) [8]. Contrast-enhancement significantly improves the delineation of tumor margins, differentiation between different pathological conditions, and evaluation of treatment response. In addition, perfusion-weighted imaging (PWI) allows for the evaluation of microvascular parameters related to blood flow within the tumor tissue [9].

The safety profile of GBCAs has been a subject of evolving clinical understanding. While these agents are generally safe, two key concerns have emerged. Initially, a primary risk was nephrogenic systemic fibrosis, which was linked to the use of linear GBCAs in patients with severe kidney issues. New cases of this rare disease have become very uncommon since the use of GBCAs was restricted in subjects with renal failure [10]. More recently, attention has shifted to gadolinium retention in the brain and other tissues related to dechelation of GBCAs (linear > macrocyclic), also in patients with preserved renal function [11, 12]. Although no negative symptoms have been directly linked to this deposition, regulatory agencies have issued guidelines to address this concern. In fact, the European Commission has restricted the use of linear GBCAs for head and neck MRIs, emphasizing judicious use of the remaining macrocyclic agents (i.e., Gadoterate, Gadobutrol, and Gadoteridol) [13]. According to the latest European Society of Urogenital Radiology guidelines, mandatory renal function testing is not required before administering macrocyclic GBCAs, but they should be used cautiously in patients with an estimated glomerular filtration rate < 30 mL/min/1.73 m² [14]. Additionally, there are increasing environmental worries linked to the mining of gadolinium and its subsequent dispersion into the ecosystem following renal excretion [15–17]. Given their high stability, GBCAs are not eliminated by standard wastewater treatment, resulting in widespread contamination with gadolinium of freshwater and drinking water. While the long-term effects of this environmental gadolinium on humans and marine life are still being studied, it is important to monitor and evaluate its impact as more information emerges [18, 19].

Based on these concerns, recommendations for the proper use of GBCAs have recently been published regarding patient categories with chronic diseases who undergo repeated contrast-enhanced MRI over their lifetime (e.g., multiple sclerosis and intracranial tumors) [20–22]. However, a comprehensive evaluation regarding GBCA usage in HNCs is still lacking.

In this narrative review, we aim to provide a framework for clinicians on the role of GBCAs in the diagnosis, staging, and monitoring of HNCs, the main sequences employed after GBCA administration, along with their interpretation and potential alternatives. In addition, it discusses future developments in hybrid imaging, novel contrast agents, and artificial intelligence (AI) tools.

## MRI sequences

When performing contrast-enhanced T1-weighted MRI of the neck, turbo- or fast spin echo and gradient-echo sequences are typically used. To effectively differentiate enhancing tissues from fat, fat suppression techniques are crucial. These commonly include spectral or frequency-selective fat suppression or chemical shift-based Dixon methods, the latter particularly useful for reducing artifacts related to magnetic field inhomogeneities by dental prostheses [8, 9]. Historically, contrast-enhanced imaging of the neck involved acquiring separate 2D images in the axial and coronal planes. However, 3D post-contrast T1-weighted imaging has now become routine. This approach offers several advantages, including thinner slices with higher spatial resolution, improved vascular assessment, and reduced total scan time [8]. Notably, a 3D post-contrast T1-weighted sequence is typically the only 3D sequence included in head and neck MRI protocols. The possibility of generating multiplanar reconstructions is particularly valuable for evaluating the tumor’s relationship with the complex regional anatomy. For example, 3D gradient-echo sequences allow for acquiring high-resolution volumetric imaging while reducing motion artifacts, without sacrificing either image clarity or the detectability of tumors [23–25]. Newer techniques, like radial imaging, now produce higher-resolution images with better overall quality and fewer artifacts than traditional T1-weighted turbo spin echo sequences [26].

PWI in HNCs is commonly obtained through dynamic contrast-enhanced (DCE) MRI. This involves acquiring a rapid series of T1-weighted gradient-echo images before, during, and after an intravenous injection of a GBCA [27]. Regions of interest are placed within the tumor to calculate various semiquantitative parameters, such as wash-in and wash-out rates (the rate at which the GBCA enters and leaves the tumor), peak enhancement (the highest concentration of contrast reached), time-to-peak enhancement (the time it takes to reach the maximum enhancement), and the area under the curve (the overall amount of enhancement present over time) [9]. Beyond semiquantitative metrics, pharmacokinetic modeling provides quantitative parameters in DCE-MRI, including: *K*^trans^ (volume transfer coefficient describing the movement between plasma and the extravascular extracellular space), *k*_ep_ (rate constant indicating the transfer rate from the extravascular extracellular space back to the plasma), *v*_e_ (the fractional volume of the extravascular extracellular space, indicating the available space within the tissue interstitium for the accumulation of GBCA), and *v*_p_ (the fractional plasma volume, reflecting the blood plasma within a given tissue volume) [9, 28]. Arterial spin labeling (ASL) has emerged as a promising non-contrast alternative to DCE-MRI for perfusion assessment in HNCs [29, 30]. In ASL, a radiofrequency pulse magnetically labels water protons in arterial blood by saturating them. By subtracting the labeled images from the control images, static background signals are removed, isolating a perfusion-dependent signal [31]. Labeling techniques include continuous, pulsed, and velocity-selective [29]; among these, pseudo-continuous ASL (pCASL) is recommended for neck imaging, using a rapid sequence of radiofrequency pulses to achieve labeling at a rate of roughly one pulse per millisecond [32]. In clinical practice, ASL in the neck is typically approximated by visually examining the subtracted images to determine where the tagged spins are distributed. This approach, however, can lead to biased and subjective evaluations because the background signal is not always optimally suppressed [33]. The most effective way to leverage ASL data involves a quantitative approach, particularly for calculating the tumor blood flow (TBF) in neck lesions [34, 35].

Table 1 summarizes the GBCA doses and contrast-enhanced MRI sequences applicable in daily clinical practice in HNCs.

**Table 1:** Summary of suggested GBCAs doses and contrast-enhanced magnetic resonance imaging (MRI) sequences applicable in daily clinical practice in HNCs

	GBCA dose and injection	Sequences		
		Suggested	Optional	Not suggested
Diagnosis	• Dose: 0.1 mmol/Kg for Gadoterate, Gadobutrol, and Gadoteridol; 0.05 mmol/Kg for Gadopiclenol; 0.04 mmol/Kg for Gadoquatrane • Injection: power injector (rate between 2 and 4 mL/s) is mandatory if DCE is performed, and it is recommended over manual injection in all other cases	3D isotropic T1-weighted with fat-saturation	• DCE for identification of neck carcinoma of unknown primary and characterization of parotid lesions • 2D T1-weighted with fat-saturation^°^	ASL
Staging and monitoring^*^		3D isotropic T1-weighted with fat-saturation	2D T1-weighted with fat-saturation^°^	• DCE • ASL

*ASL* arterial spin labeling, *DCE* dynamic contrast-enhancement, *GBCA* gadolinium-based contrast agents, *HNCs* head and neck cancers

^°^The appropriate plane is selected according to the tumor’s site and spread, with a minimum of 2 planes if 3D imaging is not performed

^*^For monitoring patients who continue to undergo follow-up MRI scans beyond 27 months post-treatment, with previous radiological scans negative for disease recurrence and no suspicious clinical findings (e.g., endoscopy negative for suspected tumor recurrence, neck palpation negative for new cervical lymphadenopathies or masses, absence of new signs or symptoms attributable to cranial nerve involvement), GBCA may be injected only when interval changes are detected on unenhanced sequences. This conditional suggestion is inferred from the Neck Imaging Reporting and Data System’s surveillance algorithm, though it awaits further prospective validation

## GBCA in the diagnosis of HNCs

From a diagnostic perspective, head and neck masses present a considerable challenge, with a broad differential that includes inflammatory, infectious, and neoplastic conditions [36].

**Fig. 1:**
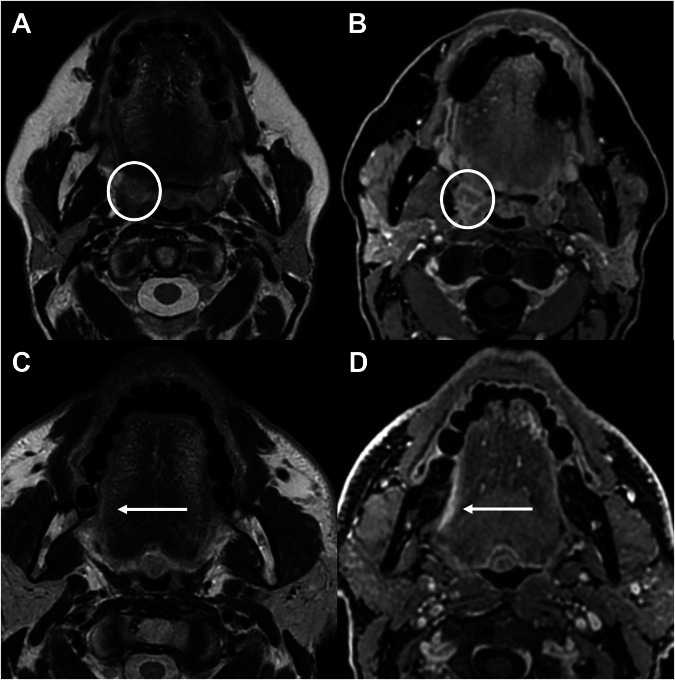
In a patient with a small right tonsillar tumor (circles in **A**, **B**) and one with a small right lateral tongue tumor (arrows in **C**, **D**), fat-saturated T1-weighted images after GBCAs administration (**B**, **D**) better identify the neoplasm compared to T2-weighted images (**A**, **C**). GBCA, gadolinium-based contrast agents

HNCs typically exhibit intermediate T2 signal intensity (higher than muscle), low T1 signal intensity, and less contrast-enhancement than normal mucosa on MRI. GBCAs improve the visibility of small solid lesions (e.g., tongue border and tonsillar regions, Fig. 1) and enable their distinction from lesions with purulent (e.g., abscess) or mainly cystic (e.g., congenital lesion) contents [9]. When examining the sinonasal cavity, contrast-enhanced MRI aids in differentiating tumors from benign secretions (Fig. S1) and is considered superior to CT in distinguishing inflammatory from neoplastic processes [37].

**Fig. 2:**
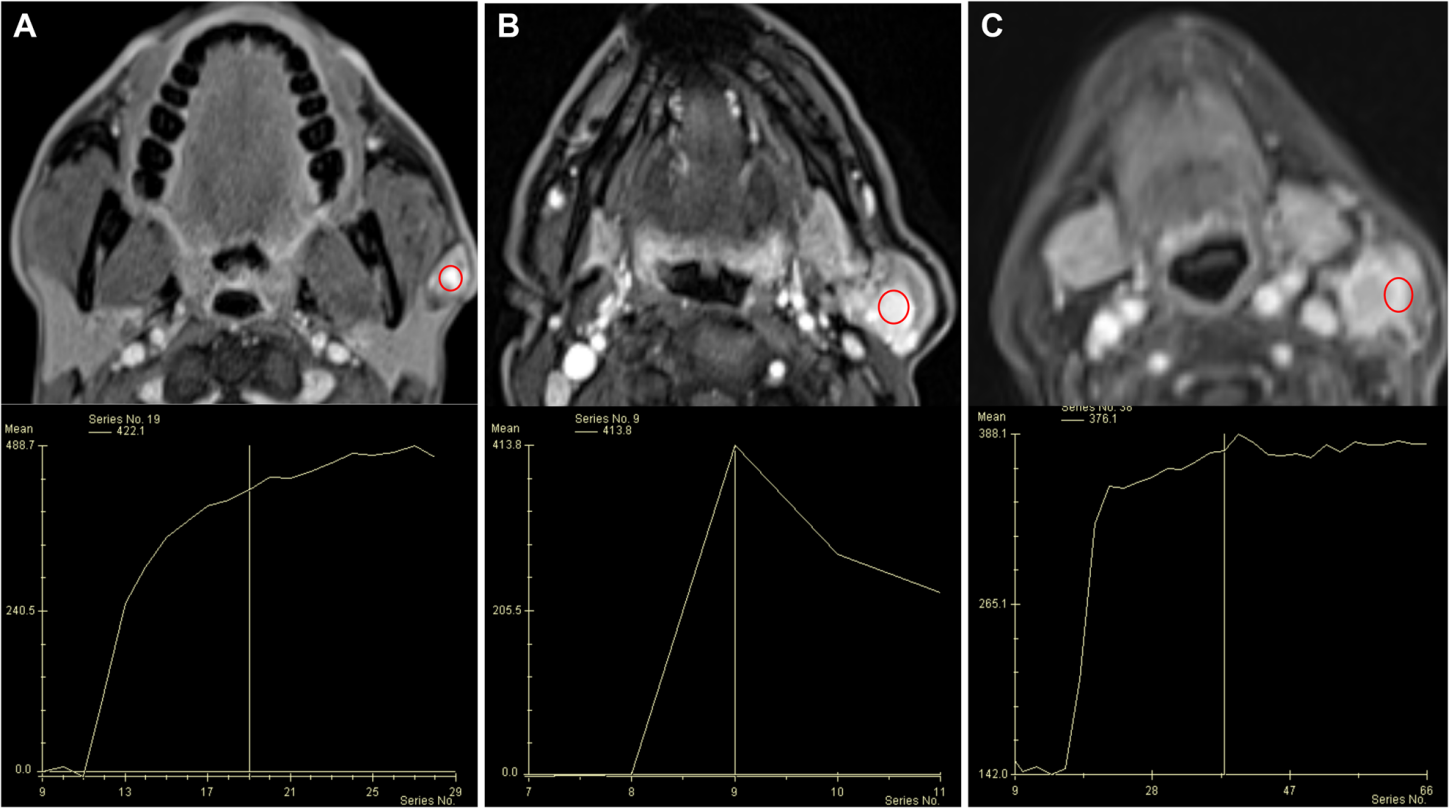
Differential diagnosis of solid left parotid lesions using dynamic-contrast enhancement and time-intensity curve analysis. The red circles represent the region of interest placed on the vascularized part of the tumor for the generation of intensity-time curves. **A** Pleomorphic adenoma with a Type A curve (prolonged time-to-peak: 150 s). **B** Warthin’s tumor with a Type B curve (short time-to-peak: 45 s, significant wash-out: > 30%). **C** Adenocarcinoma with a Type C curve (short time-to-peak: 93 s, minimal wash-out: < 30%)

DCE-MRI scans aimed at distinguishing SCC from lymphoma have employed diverse imaging protocols and yielded somewhat inconsistent outcomes. However, a general trend in the literature suggests that SCC exhibits greater tumor perfusion and capillary permeability compared to lymphoma. This is often indicated by higher *K*^trans^ and *v*_e_ values, as well as greater peak contrast-enhancement and a shorter time-to-peak on time-intensity curve analysis [38]. Furthermore, visual evaluation of early-phase DCE-MRI can reveal minute tumors not visible on conventional sequences, which may be crucial in cases of HNC of unknown primary (Fig. S2). DCE-MRI also facilitates differentiation of hypovascular tumors (e.g., schwannomas) from hypervascular tumors (e.g., paraganglioma) [28, 38]. Parotid lesions might be differentiated based on their DCE-MRI patterns using three key parameters: time-to-peak, wash-out ratio, and the shape of the time-intensity curve (Table 2 and Fig. 2) [39]. Finally, preliminary studies suggest that ASL can distinguish SCC from other types of benign or malignant tumors in regions like the paranasal sinuses, upper airway, parotid glands, and orbits by measuring differences in TBF [29, 30].

**Table 2:** Key characteristics of parotid tumors detectable by DCE-MRI

Tumor type	Time-intensity curve type	Specification
Pleomorphic adenoma	A	• Prolonged time-to-peak (> 120 s) • No wash-out • Ascending plateau
Warthin tumor	B	• Short time-to-peak (≤ 120 s) • Significant wash-out (≥ 30%)
Malignant tumors	C	• Short time-to-peak (≤ 120 s) • Minimal wash-out (< 30%) • Descending plateau
Cyst	D	No enhancement

*DCE* dynamic contrast-enhanced

Therefore, in head and neck MRI, especially in cases where HNCs are suspected, GBCAs provide vital supplementary data for a more conclusive diagnosis. While routine use of PWI is not yet standard [40], DCE-MRI can assist in visually detecting small tumors and characterizing solid parotid lesions. ASL enables the distinction between solid and cystic lesions and allows for the estimation of TBF in neoplasms, making it a compelling alternative to DCE-MRI for evaluating HNCs.

## GBCA in the staging of HNCs

**Fig. 3:**
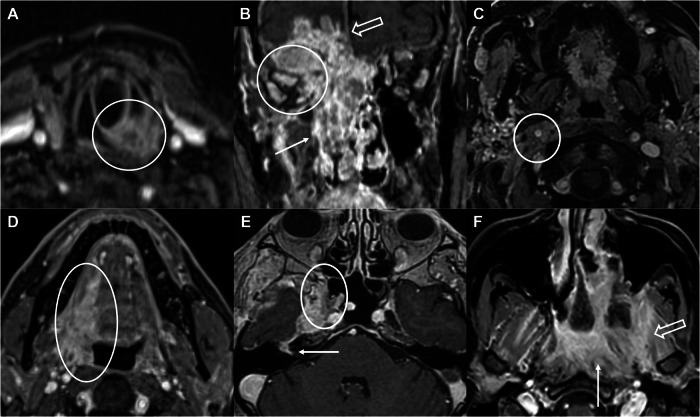
Examples of T1-weighted images after GBCA administration for defining the locoregional extent of disease. **A** Left laryngeal tumor. Both the cricoid cartilage infiltration and the extra-laryngeal extension are well-delineated (circle). **B** Extensive intracranial (empty arrow), intraorbital (circle), and ethmoido-nasal (arrow) infiltration in a sinonasal undifferentiated carcinoma. The contrast agent makes it possible to clearly distinguish the tumor tissue from the mucosal thickening in the right maxillary sinus and better delineates the infiltration of the ocular muscles and cranial cavity. **C** Encasement of the right carotid space (circle) with occlusion of the internal jugular vein in a right tonsill carcinoma. **D** Right tonsillar tumor. The extension to the oral floor and tongue is well-delineated (ellipse). **E** Nasopharyngeal carcinoma extending to the right pterygopalatine fossa (circle). The centripetal PNS along the greater superficial petrosal nerve to the geniculate ganglion and along the facial nerve in the internal auditory canal (arrow) is well-delineated. **F** Extensive infiltration of the left prevertebral (arrow) and masticator (empty arrow) spaces in a nasopharyngeal tumor. GBCA, gadolinium-based contrast agents; PNS, perineural spread

From a staging perspective, MRI is generally the preferred technique for suprahyoid disease. Conversely, contrast-enhanced CT is often the first-line choice for infrahyoid sites, which are more susceptible to motion artifacts. However, the use of MRI for the larynx and hypopharynx is becoming more common [41]. Distinguishing between an SCC and its adjacent inflammatory reaction is feasible through a detailed analysis of multiparametric MRI signal characteristics. Notably, inflammatory processes tend to show greater signal intensity on T2 sequences and enhance more robustly following GBCA administration when compared to the tumor [42]. Contrast-enhanced MRI is crucial in evaluating intraorbital or intracranial extension, commonly seen in carcinomas originating from the nasal cavity, paranasal sinuses, and nasopharynx [8]. Contrast-enhanced MRI is also the favored method for visualizing perineural spread (PNS), which is a crucial factor in predicting the outcome of HNCs [43]. PNS appears as asymmetric nerve enhancement, thickening, or obliteration of fat at neural foramina. Advanced PNS may also show bony erosion of the skull base foramina [44]. Figure 3 shows examples of contrast-enhanced images for defining the locoregional extent of HNCs.

According to the primary tumor location, specific features warrant focus during the evaluation of post-contrast imaging, as they impact accurate T staging [45–49].

PWI has been used to predict treatment responses. Despite the intricate biological interplay between tumor perfusion, permeability, and how tumors respond to chemoradiation, current findings generally suggest that tumors exhibiting good baseline perfusion and permeability, as indicated by higher *K*^trans^ values on DCE-MRI, tend to have less hypoxia. This lower hypoxia is associated with increased sensitivity to chemoradiation treatment and improved survival outcomes [38]. Moreover, as highlighted in the literature and recognized by experts, PWI may also have utility in radiotherapy planning for gross tumor volume delineation [28, 40]. ASL has shown potential for predicting treatment outcomes in HNCs, considering that lower TBF values prior to treatment have been linked to a less favorable prognosis [35].

Finally, it is important to remember that the use of GBCAs helps in delineating the structure of cervical lymph nodes, which, if involved, influence the patient’s staging. For example, contrast-enhanced MRI facilitates the identification of cystic lymph nodes, often associated with human papilloma virus-positive oropharyngeal SCC, as well as signs of extra-nodal extension [50, 51]. Irregularities at the lymph node borders may indicate extra-nodal extension, where cancer cells breach the nodal capsule. This often appears on imaging as capsular enhancement, indistinct margins, and surrounding fat stranding [45, 51]. PWI has also been tested in N staging. Lymph node metastases exhibiting good baseline perfusion and permeability, as indicated by higher *K*^trans^ values on DCE-MRI, tend to show greater sensitivity to chemoradiation and, consequently, improved survival rates. Conversely, nodal metastases with low *K*^trans^ values are generally associated with poorer prognoses [38]. Furthermore, DCE-MRI may also aid in distinguishing metastatic from benign lymph nodes by revealing reduced extracellular extravascular space volume and slower contrast transfer, potentially enhancing the accuracy of nodal staging [52].

In summary, GBCA-enhanced imaging represents a key component of multiparametric MRI analysis for T and N assessment in patients with HNCs. Additional evidence is necessary to ascertain the prognostic value of PWI (with DCE-MRI and/or ASL) in the evaluation of both the primary tumor and cervical lymph nodes.

## GBCA in the monitoring of HNCs

**Fig. 4:**
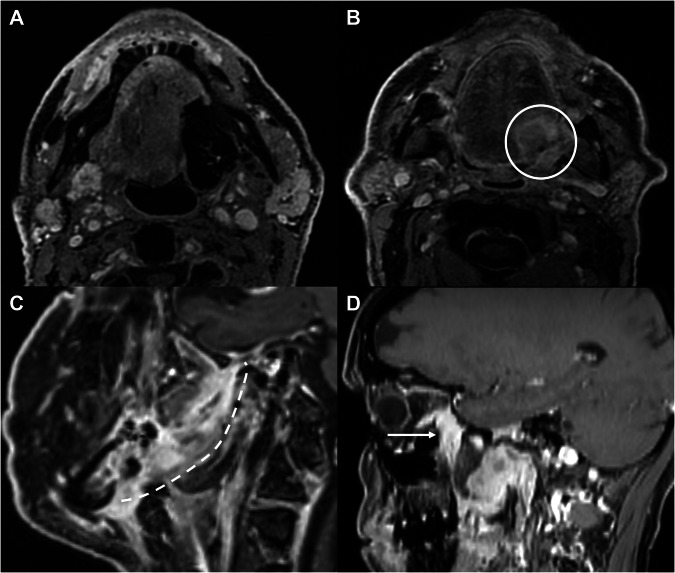
Examples of findings in T1-weighted images after GBCA administration during follow-up. **A** Left oropharyngeal tumor surgically removed with flap reconstruction, showing no evidence of macroscopic recurrence. **B** Recurrence of a left base tongue tumor (circle). **C** Recurrence of a retromolar trigone tumor along the pterygomandibular raphe (dashed line). **D** Recurrent nasopharyngeal carcinoma reaching the pterygopalatine fossa (arrow) with centrifugal PNS along the infraorbital and greater palatine nerves. GBCA, gadolinium-based contrast agents; PNS, perineural spread

From a monitoring perspective, imaging is crucial in the management of HNCs to detect any residual disease or recurrence at an early stage, thereby optimizing the potential for effective salvage therapies. Surveillance imaging protocols differ in their recommendations for timing and modality. Generally, a baseline scan is advised 3–6 months post-treatment, depending on therapy type and patient risk [53]. Distinguishing recurrent tumors from the typical tissue changes following surgery, radiation, or chemotherapy poses a major challenge in post-treatment imaging [53–56]. Contrast-enhanced MRI can be especially valuable in addressing this difficulty (Table 3). For instance, post-contrast imaging of larynx or hypopharynx cancer may assist in differentiating laryngeal cartilage invasion from inflammation, considering that inflamed but non-infiltrated cartilage shows more pronounced and earlier enhancement than that of residual tumor [22]. Figure 4 shows examples of contrast-enhanced images during follow-up of HNCs.

**Table 3:** Key post-contrast imaging features to examine during HNC surveillance

High suspicion of recurrence	
Primary site	• Nodule or mass at the primary site, particularly if new or growing, with signal characteristics and enhancement patterns consistent with the original tumor • Perineural tumor spread
Lymph nodes	New or growing lymph node that shows necrosis, irregular margins, or heightened enhancement
Main post-operative findings	
Vascularized scar	Contrast-enhancement that fades over time suggests early vascularized scar tissue, whereas chronic scar tissue is indicated by low signal on T2-weighted imaging
Flap	• The muscular component of a myocutaneous flap may show different enhancement patterns • Swollen reactive lymph nodes often appear in the mesenteric fat around a free jejunum flap
Normal tissue remnants	Contrast-enhancement in residual tongue or thyroid tissue could be misinterpreted as a recurrence
Main post-operative complications	
Seroma	Fluid collection without contrast-enhancement
Abscess	Fluid collection with diffusion restriction and rim-enhancement
Flap necrosis	Thrombosis of neck vessels and a non-enhancing flap
Main early changes after radiotherapy/chemoradiotherapy	
Mucositis	Swelling of the pharynx and larynx with contrast-enhancement of their walls
Sialadenitis	Swelling of the submandibular and parotid glands with contrast-enhancement
Thyroiditis	Swelling of the thyroid gland with heterogeneous contrast-enhancement
Main post-radiotherapy complications	
Mucosal necrosis	Absence of mucosal enhancement with or without ulceration
Trismus	Pterygoid muscle atrophy with enhancement confined to the radiation field
Osteoradionecrosis	Abnormal marrow signal and widespread, strong contrast-enhancement, with no associated soft tissue mass. Unenhanced areas within the bone point to sequestrum formation in osteomyelitis
Chondroradionecrosis	Contrast-enhancement of collapsed thyroid cartilage
Vascular complications	Internal jugular vein thrombosis or mural thickening of the carotid artery
Neurological complications	• Radiation-induced temporal lobe necrosis appears as a ring-enhancing lesion surrounded by perilesional edema • Cranial and brachial neuropathy are characterized by thickening and enhancement of the affected nerves, and their roots and trunks, respectively • Acute radiation-induced spinal cord injury manifests as an enlarged, T2 hyperintense cord with contrast-enhancement

To ensure consistent interpretation and reporting of surveillance imaging, the Neck Imaging Reporting and Data System (NI-RADS) was developed by the American College of Radiology [57], originally for CT but now adapted for MRI [58–61]. The use of GBCAs is essential for evaluating the primary site and the neck (nodal assessment), which is necessary for assigning all NI-RADS categories [62]. Moreover, according to the NI-RADS authors, contrast-enhanced sequences should be acquired in a minimum of 2 planes, and imaging surveillance could conclude 27 months after treatment if all radiological follow-ups have consistently been negative [54, 59].

PWI has been investigated in three distinct scenarios for patients undergoing treatment for HNCs: early response assessment during chemo-radiotherapy, response evaluation after chemo-radiotherapy or induction chemotherapy, and detection of local tumor recurrence. Regarding treatment response evaluation, tumors showing good perfusion on DCE-MRI following initial therapy (high blood volume and high *K*^trans^ values) that decline during therapy tend to show better local control [28, 38, 40]. For identifying local tumor recurrence, DCE-MRI may offer greater specificity compared to conventional imaging by quantitatively characterizing the changes in enhancement over time. Residual tumor tissue, in particular, enhances earlier and more intensely than benign post-treatment tissue [28, 38, 40]. This is consistent with the common subjective observation that fibrotic tissue shows delayed and less avid contrast-enhancement on routine scans [63]. Finally, ASL has revealed a significant increase in TBF in patients with recurrent HNCs when compared to those without residual tumor or exhibiting post-radiation changes [35, 64, 65].

In summary, GBCA administration is a necessary component of the multiparametric MRI analysis for individuals under surveillance for HNCs. Patients undergoing long-term MRI follow-up (beyond 27 months) with no clinical or radiological signs of recurrence may be considered for non-contrast scans. The use of GBCA could be reserved for cases where unenhanced sequences show new changes. This conditional suggestion is inferred from the NI-RADS’ surveillance algorithm, though it awaits further prospective validation. PWI (with DCE-MRI and/or ASL) has shown encouraging results in evaluating therapy response and in distinguishing between disease recurrence and treatment-related effects.

## Future directions

### Positron emission tomography (PET)/MRI

Contrast-enhanced MRI and PET should not be viewed as mutually exclusive techniques but rather as complementary tools for the comprehensive evaluation of HNCs [66]. MRI offers superior anatomical resolution, while PET allows for whole-body assessment, making it the most effective tool for detecting nodal involvement, distant metastases, and the potential presence of synchronous tumors [67]. Moreover, PET can demonstrate an absence of tumor activity even before morphological changes are evident on MRI. However, PET can show uptake in various benign processes, including benign tumors, inflammatory conditions, post-traumatic changes, and iatrogenic effects [68]. Hybrid PET/MRI systems have emerged, combining the benefits of both PET and MRI. Nevertheless, the precise role of this integrated technique in the diagnosis, treatment planning, and follow-up of HNCs is still being determined [8]. A 2020 study indicated that GBCA administration might not offer additional benefits for local tumor staging in PET/MRI for HNCs. However, the authors recommended retaining post-contrast sequences for salivary gland malignancy work-ups due to their high propensity for nerve invasion, or when PNS is clinically suspected [69].

#### Novel contrast agents

Considering the concerns about gadolinium deposits and their environmental impact, there is increasing momentum toward developing alternative contrast agents and implementing dose-reduction strategies [11, 12].

Gadopiclenol [70, 71] and gadoquatrane [72–76] are novel macrocyclic GBCA, distinguished by their high relaxivity. They can produce diagnostic images comparable in quality to other GBCAs but with a lower dose (0.05 mmol/kg for gadopiclenol, 0.04 mmol/kg for gadoquatrane). Gadopiclenol was approved by the European Medicines Agency in December 2023 [70, 71], while gadoquatrane was recently submitted to the Food and Drug Administration for approval.

RVP-001 is a novel contrast agent for MRI in clinical development in patients with lesions of the central nervous system. Its mechanism of action harnesses manganese, an element essential for life and naturally present in the human body, suggesting a superior safety profile compared to gadolinium [77].

Molecular imaging of HNCs employs contrast agents across multiple modalities that bind specific tumor-associated molecular markers, all currently in preclinical development. Examples for MRI include peptide-guided gadolinium compounds (e.g., MT218 targeting extradomain-B fibronectin, overexpressed in aggressive tumors) for precise localization and characterization; manganese-based probes (e.g., AZA-TA-Mn targeting carbonic anhydrase IX in hypoxic regions) for detailed hypoxia imaging critical to assessing tumor aggressiveness and treatment resistance; and functionalized nanoparticles (e.g., biotin/PEG-UCNPs, single-atom Gd nanospheres, NaHoF₄) [78].

#### AI tools

AI models, including convolutional neural networks and deep learning algorithms, are showing great promise in interpreting MRI scans for HNC diagnosis [79].

One key application is radiomics, where AI models can extract vast amounts of quantitative data from MRI sequences (like contrast-enhanced T1-weighted images) to predict treatment responses, such as the effectiveness of induction chemotherapy in nasopharyngeal carcinoma [80, 81]. AI also holds potential for early detection of treatment toxicity, like predicting radiation-induced brain injury before symptoms appear [82]. Despite these exciting advancements, prospective, multicenter validation and standardization of radiomic methods are advised before they can be used routinely in clinics.

AI algorithms can simplify GBCA PWI of HNCs by automating post-processing and initially quantifying parameters, such as those used to differentiate tumor from treatment effects [83].

Finally, machine learning methods have been applied to reduce or eliminate GBCA administration, achieving diagnostic performance comparable to true gadolinium-enhanced scans [84, 85]. Although most work to date has focused on brain, spine, and abdominal imaging, these “virtual contrast” approaches could eventually be adapted for head and neck MRI, obviating or dramatically reducing gadolinium use. Future research should therefore prioritize multicentre validation of AI tools, specifically tailored to the anatomical challenges of HNCs.

In summary, future research needs to investigate PET/MRI to determine in which cases GBCA administration can be avoided. New GBCAs promise to provide the same diagnostic information as macrocyclic agents but at lower doses, while novel gadolinium-free contrast agents are also being explored specifically for use in HNCs. Finally, AI tools could be beneficial in extracting radiomic information, in enhancing the analysis of PWI, and in reducing the dose of GBCA injected.

## Conclusion

GBCA-enhanced imaging remains a fundamental component of multiparametric MRI for diagnosis, staging, and monitoring HNCs, typically using a 3D T1-weighted sequence with fat saturation at equilibrium. PWI shows promise for managing HNCs, but it needs further standardization before it can be routinely used in clinical practice. ASL is proving to be an interesting alternative for this purpose, offering the significant benefit of not requiring GBCA administration. Hybrid imaging with PET/MRI, the use of novel contrast agents, and AI tools are all promising options for reducing or eliminating GBCA use. More research is needed to improve these alternative methods and make them more suitable for widespread use in clinics.

## Supplementary information

ELECTRONIC SUPPLEMENTARY MATERIAL

## Abbreviations

AI: Artificial intelligence
ASL: Arterial spin labeling
CT: Computed tomography
DCE: Dynamic contrast-enhanced
GBCA: Gadolinium-based contrast agent
HNC: Head and neck cancer
MRI: Magnetic resonance imaging
NI-RADS: Neck imaging reporting and data system
PET: Positron emission tomography
PNS: Perineural spread
PWI: Perfusion-weighted imaging
SCC: Squamous cell carcinoma
TBF: Tumor blood flow
