# Supplementary material for: Use of gadolinium-based contrast agents in head and neck cancer diagnosis, staging, and monitoring: current applications and future perspectives
Source: Eur Radiol. 2025 Dec 13;36(5):3648–60. doi: 10.1007/s00330-025-12165-0 (PMC13086760; doi:10.1007/s00330-025-12165-0)
Supplement: Supplementary file 1 — ELECTRONIC SUPPLEMENTARY MATERIAL [file 330_2025_12165_MOESM1_ESM.pdf]

Use of gadolinium-based contrast agents in head and neck cancer diagnosis, staging and monitoring: current applications and future perspectives

ELECTRONIC SUPPLEMENTARY MATERIAL

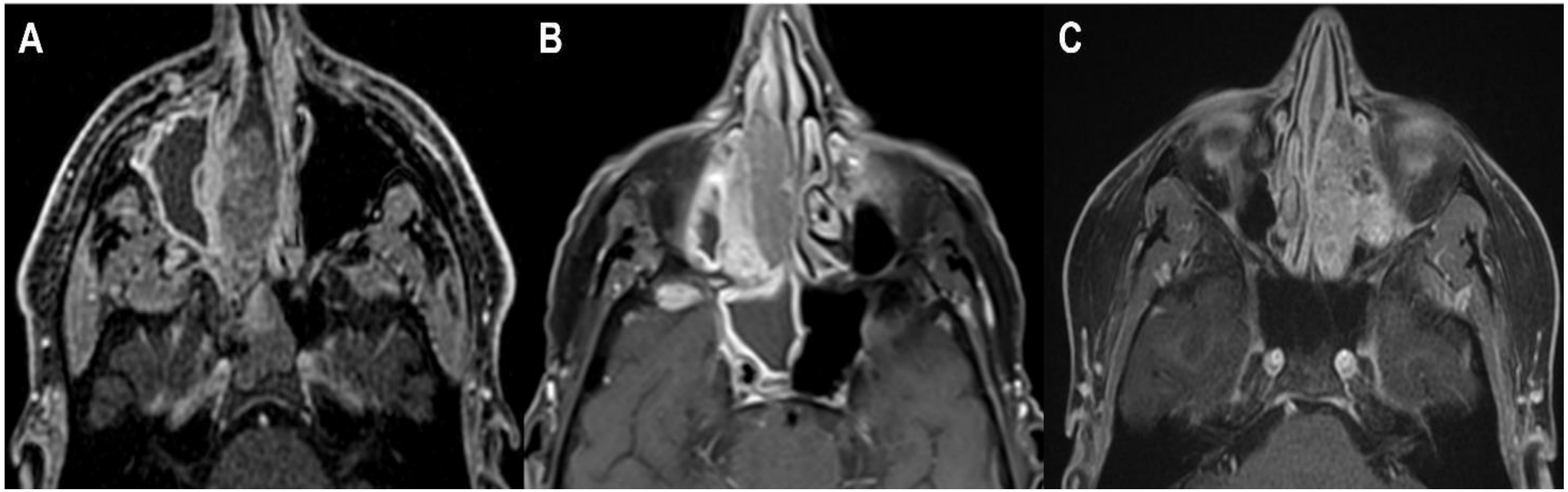

Figure S1. Fat-saturated T1-weighted images after gadolinium-based contrast agent administration in a patient with squamous cell carcinoma (A), adenocarcinoma (B), and neuroblastoma (C) developing in the nasal fossae. Note that although the neoplasms in A and B are less vascularized compared to C, enhancement characteristics show significant overlap among different malignancies, which limits tissue characterization based on enhancement alone. Furthermore, in the post-contrast images in A and B, the difference between the inflamed paranasal sinus mucosa and the tumor mass is clearly visible.

Eur Radiol (2025) Parillo M, Vaccarino F, Falzone A, et al.

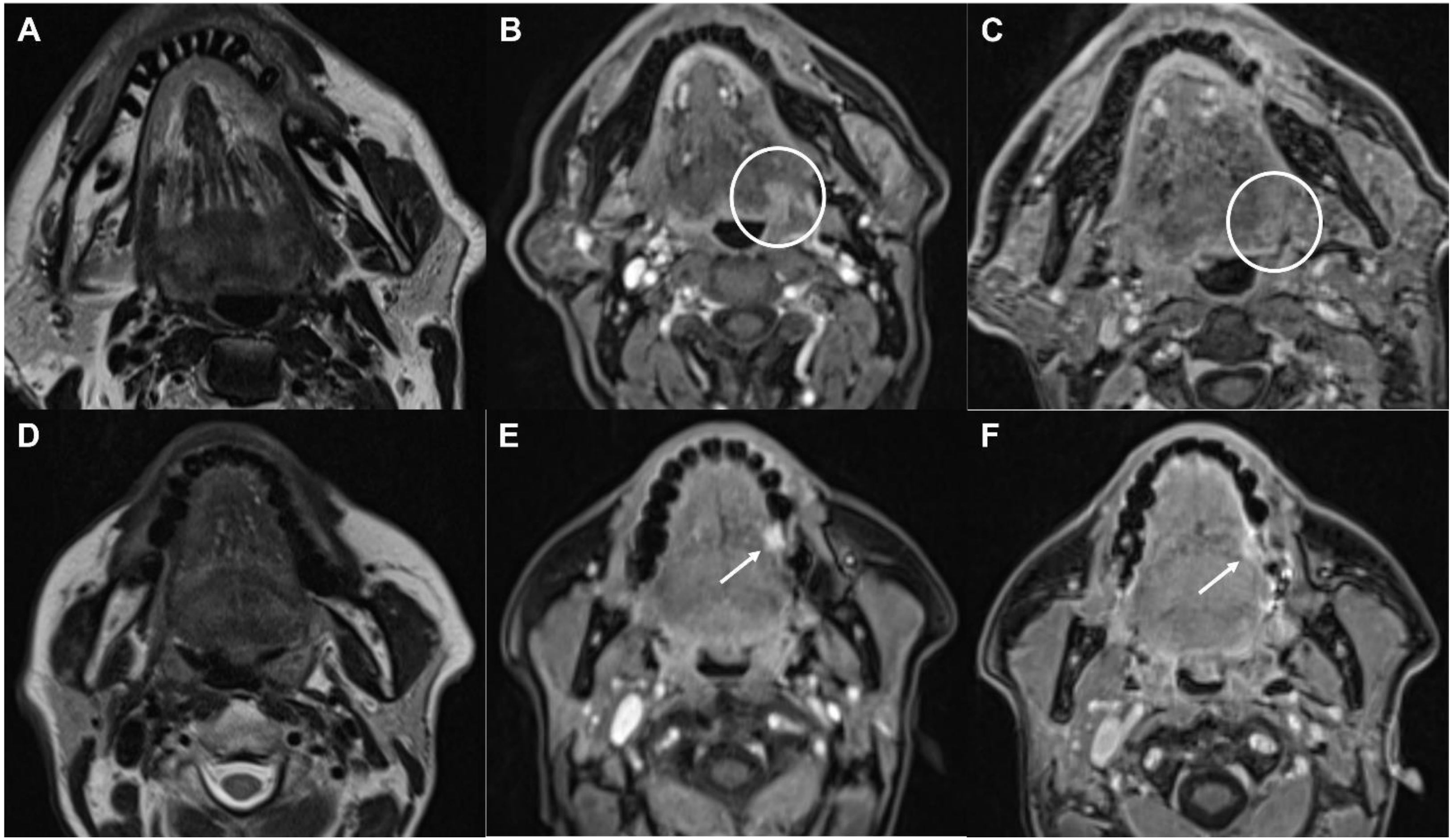

Figure S2. Identification of small lesions through visual assessment of dynamic-contrast enhancement. In the first patient (A, B, C) a left base tongue tumor (circles) is visible on early postcontrast image (B) but not on late one (C) or T2-weighted image (A). In the second patient (D, E, F) a left lateral tongue tumor (arrows) is better visible on early post-contrast image (E) than in late one (F) or T2-weighted image (D).
